# Pan-tissue transcriptomic profiling of dairy cattle

**DOI:** 10.1186/s40104-026-01440-9

**Published:** 2026-06-14

**Authors:** Jinfeng He, Ruike Jia, Aixia Du, Weijie Zheng, Bo Han, Qi Zhang, Zijiao Guo, Yanan Liu, Yali Hou, Dongxiao Sun

**Affiliations:** 1 State Key Laboratory of Animal Biotech Breeding, National Engineering Laboratory for Animal Breeding, Key Laboratory of Animal Genetics, Breeding and Reproduction of Ministry of Agriculture and Rural Affairs, College of Animal Science and Technology, China Agricultural University, Beijing, 100193 China; 2 State Key Laboratory of Animal Biotech Breeding, Institute of Animal Science, Chinese Academy of Agricultural Sciences, Beijing, 100193 China

**Keywords:** Alternative polyadenylation, Alternative splicing, Cross-species analysis, Dairy cattle, Non-coding RNAs, Pan-tissue transcriptomic profile

## Abstract

**Background:**

Milk production in dairy cattle is a paradigmatic complex trait emerging from coordinated regulatory programs across multiple tissues and molecular layers, while previous transcriptomic studies have largely focused on a limited number of key tissues, most notably the mammary gland and liver. Consequently, how transcriptional regulatory mechanisms underlying milk traits are coordinated across the whole organism remains poorly understood. In particular, the contributions of non-coding RNA, alternative splicing and alternative polyadenylation vary across tissues and contribute to tissue-specific regulatory landscapes has not been comprehensively profiled at a pan-tissue scale. This study aims to systematically characterize tissue-specific transcriptomic and post-transcriptional profiles across multiple tissues in dairy cattle.

**Results:**

We generated RNA-seq data for 99 tissues from two adult Holstein cows and integrated these with 400 publicly available RNA-seq samples from 182 adult Holstein cows covering 127 tissues, yielding a profiling that spans 166 tissues. Using a one-versus-all framework, we identified the tissue-specific genes thereby revealing distinct tissue metabolic demands and physiological specialization, with highly active tissues (brain, mammary gland and reproductive organs) harboring larger numbers of tissue specific genes. Further, we predicted potential RNA–RNA interactions and found that tissue-specific genes may be associated with coordinated non-coding RNA interaction networks. At the post-transcriptional level, rMATS profiling revealed skipped exons and mutually exclusive exons as the predominant alternative splicing classes across tissues, whereas proximal polyadenylation site usage was widespread, although brain-expressed genes more frequently used distal sites. Cross-species analysis based on 11,547 one-to-one orthologues shared among cattle, humans, and pigs showed that tissues from the same organ or system of cattle, humans and pigs generally clustered together, whereas the oviduct displayed expression patterns more similar to tissues within the hypothalamic-pituitary-gonadal axis.

**Conclusions:**

This study establishes an integrative multi-tissue transcriptomic and post-transcriptional regulatory profile for dairy cattle across a broad range of organs, providing a valuable resource for investigating tissue-specific variation in transcriptional and post-transcriptional regulation.

**Supplementary Information:**

The online version contains supplementary material available at 10.1186/s40104-026-01440-9.

## Background

Dairy cattle plays a vital role in global food security and human nutrition, producing nearly 80% of the world’s commercial dairy products [1]. Milk and its derivatives provide essential nutrients, including high-quality proteins, lipids, vitamins, and minerals, which are indispensable for human health and development. Whereas, major economic traits in dairy cattle, such as milk yield and milk compositions, are typical quantitative traits controlled by numerous genes and intricate regulatory networks. Deciphering the genetic mechanisms of such traits in dairy cattle and pinpointing causal SNPs/QTLs provides biological priors for molecular breeding technologies such as genomic selection (GS) and gene editing.

Transcriptomic analysis has emerged as one of the most powerful approaches for uncovering the molecular foundations of complex traits. In recent years, multi-tissue and cross-organ studies have paid more attention to revealing that physiological traits are often shaped by regulatory interactions among tissues and organs rather than by isolated tissue functions. For instance, neuroendocrine signaling has been shown to coordinate lactation and metabolism across distant organs in mammals [2–9]. Our previous study found nerve cells were associated with milk production traits through correlating single-cell gene expression from Cattle Cell Atlas (CattleCA) [10] with GWAS data of 55 complex traits in Holstein. Liu et al. [11] integrated 7,180 RNA-seq samples and GWAS from 27,214 dairy cattle, linking regulatory variants across 23 tissues to 43 traits, with liver regulatory signals showing relevance to protein-yield variation. Zhang et al. [12] identified tissue-specific genes and found that upregulated muscle oxidative metabolism and hepatic oxidative phosphorylation may enhance beef-related functions by generating an integrative transcriptome across 51 tissues from 3 beef cattle. Teng et al. [13] constructed a multi-tissue PigGTEx atlas and found that tissue-specific regulatory signals of *ABCD4* in the small intestine and brain were associated with backfat thickness. Thus, accumulating evidence suggests that complex traits result from coordinated gene expression across multiple tissues, rather than being solely determined by a single tissue. Therefore, integrating multi-tissue and cell-type–resolved transcriptomic data with genetic information can help us better understand the regulatory mechanisms underlying complex traits.

Beyond transcriptional regulation, post-transcriptional mechanisms including non-coding RNAs (ncRNAs), alternative splicing (AS) and alternative polyadenylation (APA) have been shown to play crucial roles in shaping translational efficiency, protein isoform diversity and transcript stability, providing additional regulatory layers for complex trait formation in mammals [14–17].

Over the past decades, extensive transcriptome studies in cattle have provided valuable insights into gene expression and regulation across specific biological processes. However, most of them merely focused on single or a few tissues, including mammary gland [18–20], liver [4, 21–23], embryo [24], milk [18, 25], jejunum [26], rumen epithelium [19] and testis [27].

**Fig. 1:**
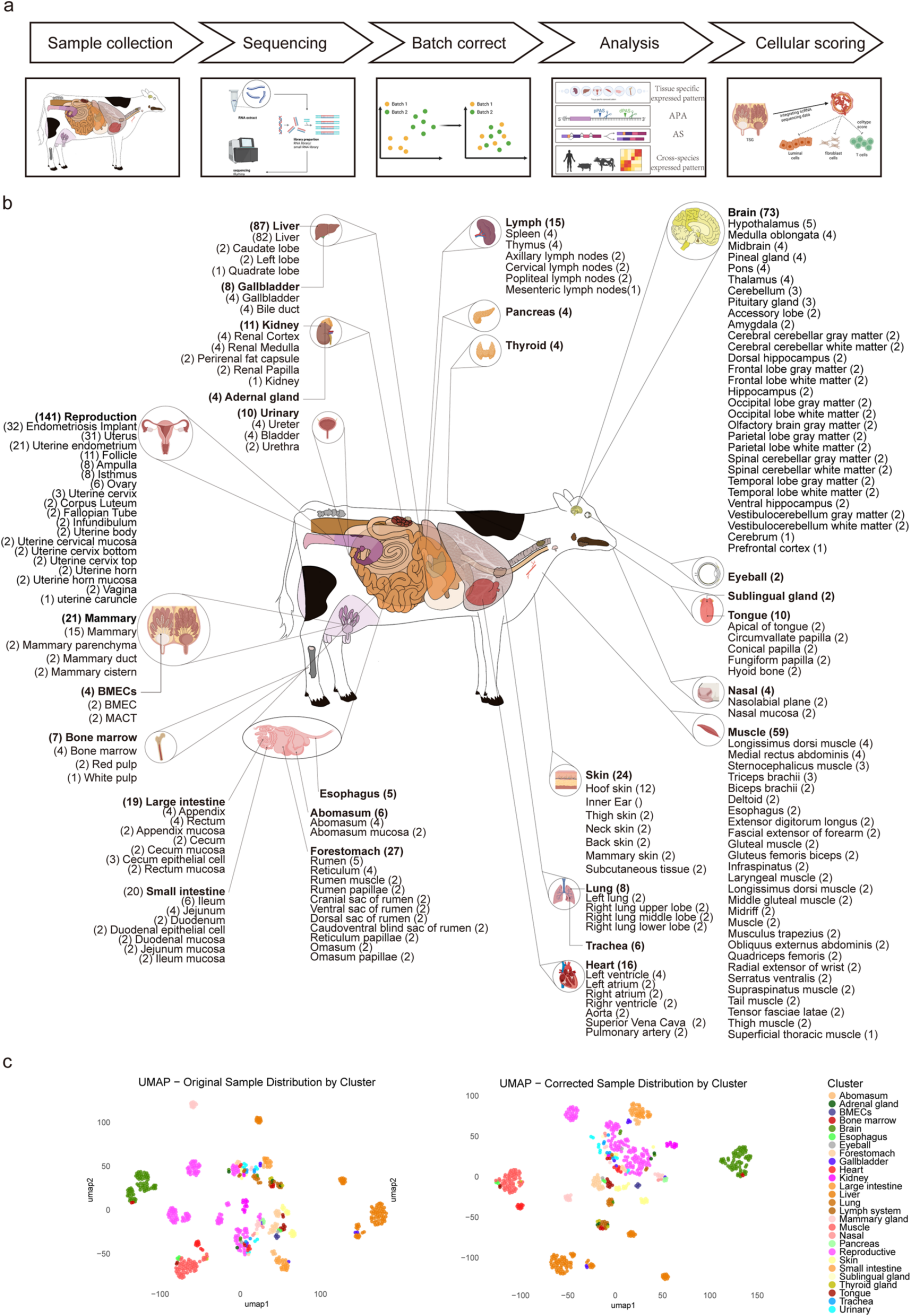
Pan-tissue transcriptomic profiling of dairy cattle. **a** Overview of the study design, analytical framework, and downstream applications of the pan-tissue transcriptomic profile. **b** Schematic summary of the 166 tissue types included in this study across 11 major physiological systems, integrating newly generated and publicly available RNA-seq datasets. Numbers in parentheses indicate the number of samples per tissue. **c** UMAP visualization of transcriptomic profiles before and after batch effect correction. Data were corrected using ComBat to adjust for the effects of sequencing platform and nation of origin

In the present study, to dissect the cross-tissue coordinated regulation of complex economic traits, we constructed a pan-tissue transcriptomic profile for dairy cattle comprising 99 tissues newly sequenced and 102 tissues from public datasets, and identified tissue specific genes across tissues to delineate tissue-level “functional fingerprints”. Furthermore, we interrogated post-transcriptional regulation analysis from three angles including ncRNAs, alternative splicing and alternative polyadenylation to map regulatory mechanisms across tissues, and cross-species comparisons among cattle, humans and pigs to reveal species-specific adaptations and tissue-biased divergence (Fig. 1a). Together, this profile serves as a multi-tissue functional genomics framework that informs cattle genetics, breeding, nutrition, disease biology, and comparative physiology.

## Materials and methods

### Animals and samples

To minimize environmental and genetic confounding, two clinically healthy, first-lactation Holstein cows from the same sire family, matched for birth and calving dates and lactation performance, were selected at the Beijing Dairy Cattle Center (Beijing, China) and maintained under standardized feeding and management conditions. Following a 12-h fasting period, animals were euthanized and 99 tissue samples were collected from each cow (Fig. 1b and Table S1). Standardized anatomical sampling was applied: bilaterally symmetric organs were sampled from the left side; large solid organs (e.g., uterine horn and kidney) from the central parenchyma; hollow organs (e.g., rumen) from the mid-ventral region; and small organs (e.g., adrenal gland) were collected in entirety. In total, 99 tissue types were obtained with two biological replicates per tissue. Liver samples were parenchymal, and mammary gland samples from the right rear quarter included parenchyma (one-third depth), ductal epithelium and lumen wall (one-half depth), and skin ~5 cm from the teat. All samples were immediately placed into 2-mL cryovials and flash-frozen in liquid nitrogen for RNA and small RNA sequencing.

In addition, raw FASTQ files of 400 publicly available Holstein cow RNA-seq datasets were downloaded from the NCBI BioProject repository based on selection criteria: adult healthy animals (including 42 primiparous and 142 multiparous cows), paired-end sequencing, at least 20 samples by the same sequencing platform (Illumina Hiseq 2000, 2500, 3000, X5, Novaseq 6000 and Nextseq 500) from the same country (Europe, China and the United States), at least two sequencing platforms from the same country. Corresponding metadata are summarized in Table S1.

Totally, this study generated RNA-seq data for 99 tissues from two adult Holstein cows and integrated these with 400 publicly available RNA-seq samples from 182 adult Holstein cows covering 127 tissues, yielding profiling spanning 166 tissues.

### mRNA sequencing and data processing

#### RNA extraction and quality assessment

Approximately 80 mg of each tissue was homogenized with a steel bead, and total RNA was extracted using TRIzol Reagent (Invitrogen, Carlsbad, CA, USA). RNA purity was assessed by spectrophotometric measurement of the A_260_/A_280_ ratio, and integrity was evaluated by 1.0% agarose gel electrophoresis and an Agilent 2100 Bioanalyzer (Agilent Technologies, Santa Clara, CA, USA) to obtain RNA integrity numbers (RIN) (Table S1). Only samples with A_260_/A_280_ ratios of 2.0–2.2 and RNA integrity number ≥ 6.0 were used for sequencing.

#### Library construction and Illumina sequencing

For each sample, 3 μg of total RNA was used to enrich poly(A)⁺ mRNA with oligo (dT) magnetic beads, followed by fragmentation, first- and second-strand cDNA synthesis, end repair, A-tailing, adapter ligation, size selection and PCR amplification. Library concentration and size distribution were assessed using a Qubit v2.0 fluorometer and an Agilent 2100 Bioanalyzer, and effective library molarity was determined by qPCR. Paired-end 150-bp sequencing was performed on an Illumina NovaSeq 6000 platform (CapitalBio Technology, Beijing, China). Sequencing data were used for mRNA, lncRNA, and circRNA analyses.

#### Gene-level quantification

To perform quality control and quantify gene expression levels for the RNA-seq data from all 166 tissues, we downloaded the bovine reference genome ARS-UCD1.2 and annotation (Bos_taurus.ARS-UCD1.2.109.gtf.gz) from Ensembl v114. Raw paired-end reads were quality-trimmed with Trim Galore v0.6.10 (-q 20 --phred33 --length 36 -e 0.1 --stringency 3 --paired --gzip --fastqc) and aligned to the genome using HISAT2 v2.2.1; alignments were sorted with SAMtools v1.19.1. Gene-level expression was quantified from uniquely mapped reads using StringTie v2.1.7 and normalized to transcripts per million (TPM). On the other hand, putative lncRNAs were identified from transcripts ≥ 200 nt with ≥ 2 exons (class codes “u”, “i”, “x”, “o”, or “j”) using PLEK v2, LGC v1.0, and CNCI. The quantification of lncRNAs was performed using the feature_count.py script. Circular RNAs were detected by realigning unmapped reads split into anchor segments to identify back-splice junctions, and circRNA identification and quantification were performed using the find_circ script.

After stringent quality control, a total of 63.76 billion clean paired-end reads were retained, with an average of 106.63 million reads per sample. Clean reads were aligned to the bovine reference genome (ARS-UCD1.2), yielding a high average unique mapping rate of 89.57% across all samples (ranging from 60.84% in follicle to 95.43% in vestibulocerebellum white matter) (Table S1).

#### Data visualization

Batch effects of sequencing platform and sample geographic origin were corrected using ComBat (sva v.3.52.0). UMAP (v.0.2.10; n_neighbors = 30, min_dist = 2.0, metric = *euclidean*, spread = 5.0, n_epochs = 3,000) was used as a post-correction quality-control visualization to detect residual batch structure. Samples that were clearly separated from the main cluster and whose separation was concordant with sequencing platform or geographic origin were considered residual technical outliers and removed. Data visualization was performed using UMAP, and gene expression heatmap was performed using ComplexHeatmap v.2.20.0.

#### Tissue-specific gene identification

Differential expression analyses across tissues were conducted using limma (v.3.60.6). Genes significantly upregulated (log_2_FC > 1; adjusted *P* < 0.05, Benjamini–Hochberg correction) in a given tissue compared to all others were defined as tissue-specific genes.

#### Cell-type scoring of tissue-specific genes using single-cell transcriptomic data

To obtain cell-type-resolved expression profiles, we used the bovine multi-tissue single-cell RNA-seq atlas [10] and performed deconvolution analysis to infer cell-type-level expression patterns. We then applied the AddModuleScore function in the Seurat package to calculate cell-type module scores for tissue-specific genes in each cell subtype. This approach allowed us to determine which cell types predominantly expressed tissue-specific gene signatures and therefore most likely contributed to tissue-specific biological functions.

#### Tissue module score calculation

To evaluate the contribution of each tissue to milk production trait, the list of 990 functional genes that have been reported associated with five milk traits (milk yield, fat yield, fat percentage, protein yield, and protein percentage) were downloaded from the Cattle QTLdb [28]. Then, tissue module score was calculated based on the expression levels of these genes across 598 tissue samples using the AddModuleScore function in Seurat (v.5.0). A score significantly different from 0.0 (*P* < 0.01) indicates significant enrichment or depletion of the selected gene set. A significantly positive score indicates relative overexpression of trait-associated genes, suggesting that the corresponding tissue or cell subtype may be more strongly associated with the milk trait.

#### Tissue‐specific lncRNA identification and cis-target prediction

Differential expression analysis was performed using limma to identify tissue‐specific lncRNAs. LncRNAs significantly upregulated (log_2_FC > 1; adjusted *P* < 0.05, Benjamini–Hochberg correction) in a given tissue relative to all others were defined as tissue-specific lncRNAs. Protein‐coding genes located within ±100 kb of each tissue-specific lncRNA were defined as putative *cis*‐regulated target genes.

#### Tissue‐specific circRNA identification and host gene annotation

Differential expression analysis was performed using DESeq2 (v1.44.0) to identify tissue-specific circRNAs. Given the typically low expression levels of circRNAs, a more lenient threshold of log_2_FC > 0.5 and Benjamini–Hochberg-adjusted *P* < 0.05 was applied. Each circRNA was then unambiguously assigned to its host gene by mapping its 5′ and 3′ splice sites to the reference annotation.

### Small RNA sequencing and data analysis

#### Library preparation and sequencing

Small RNA (sRNA) libraries were constructed from 3 µg total RNA using the NEBNext^®^ Multiplex Small RNA Library Prep Set for Illumina^®^ (New England Biolabs, Cambridge, Massachusetts, USA). Briefly, 3′ and 5′ adapters were sequentially ligated, followed by reverse transcription and PCR amplification. cDNA libraries were size‐selected by 6% polyacrylamide gel electrophoresis (PAGE) to isolate ~140 bp fragments (corresponding to ~22 nt miRNAs with adapters) and sequenced in single‐end mode on an Illumina NovaSeq 6000 platform (CapitalBio Technology).

#### miRNA expression analysis

Raw reads were quality-checked using FastQC (v.0.12.1), adapter-trimmed with Cutadapt (v.1.18) to retain 17–35 nt reads, and further quality-filtered using Trim galore (v.0.6.10). Clean reads were converted to FASTA format and dereplicated using the miRDeep2 (v.0.1.2) collapse_reads_md.pl script. Known non–miRNA sRNAs (including rRNA, tRNA, snRNA, and other sRNAs) were removed by BLASTN-short searches against Rfam. Remaining reads were aligned to exon and intron indices of the ARS-UCD1.2 bovine genome using Bowtie (v.1.3.1) to identify miRNAs, and miRNA expression levels were quantified with miRDeep2.

#### Tissue‐specific miRNA (TSM) identification and target prediction

Tissue‐specific miRNAs were identified using DESeq2 (v.1.44.0) based on raw counts from 194 samples, applying thresholds log_2_FC > 1 and Benjamini–Hochberg-adjusted* P* < 0.05. miRNA-target interactions were predicted using RNAhybrid (v.2.2.1) and miRanda (v.3.3.a).

### MiRNA and RNA target prediction and ceRNA network construction

To construct the ceRNA regulatory network, we used RNAhybrid and miRanda software to predict the targeting interactions among tissue specific mRNAs, lncRNAs, miRNAs and circRNAs. Based on the common prediction results from both RNAhybrid and miRanda, we constructed a miRNA-centered ceRNA regulatory networks, which reveal the interactions and competitive regulatory relationships between miRNAs and mRNAs, lncRNAs, circRNAs.

### KEGG enrichment analysis

Kyoto Encyclopedia of Genes and Genomes (KEGG) pathway enrichment analysis was performed for tissue-specific genes, as well as the target genes for tissue-specific circRNAs, lncRNAs and miRNAs, using the clusterProfiler R package (v.4.12.6). Pathways with an adjusted *P* < 0.05 were considered significantly enriched, revealing the biological processes that underlie tissue-specific functions.

### Alternative splicing analysis

Clean reads were aligned to the bovine reference genome (ARS-UCD1.2) with HISAT2 (v.2.2.1), and alternative splicing events were identified using rMATS (v.4.2.0) with the ARS-UCD1.2 v109 annotation. Five canonical alternative splicing types—skipped exon (SE), alternative 5′ splice site (A5SS), alternative 3′ splice site (A3SS), mutually exclusive exons (MXE), and retained intron (RI)—were quantified. Tissue-specific alternative splicing events were detected by comparing each tissue against all others, with percent spliced-in (PSI) calculated for each event. Differential splicing was defined by a percent spliced-in difference ≥ 0.1 and Benjamini–Hochberg-adjusted False Discovery Rate < 0.05.

### Alternative polyadenylation analysis

Variable polyadenylation sites were identified using DaPars (v.2.0) to construct an alternative polyadenylation event library. Polyadenylation sites usage was quantified with QAPA (v.1.3.3), which estimates distal versus proximal poly(A) site usage for each gene. To ensure robustness, only polyadenylation sites with site usage > 10% in at least 2 samples were retained.

### Cross-species analysis

RNA-seq data comprising 58,988 samples across 35 tissues from the Human GTEx project and 31,908 samples across 22 tissues (2–1,321) from the Pig GTEx database were collected. One-to-one orthologous genes shared among humans, pigs and cattle were identified and used for cross-species comparative analyses and heatmap construction. For cross-species correlation analyses, gene expression matrices were rebuilt based on one-to-one orthologous genes to ensure comparability of gene expression profiles across species.

## Results

### Landscape of gene expression patterns across 166 bovine tissues

**Fig. 2:**
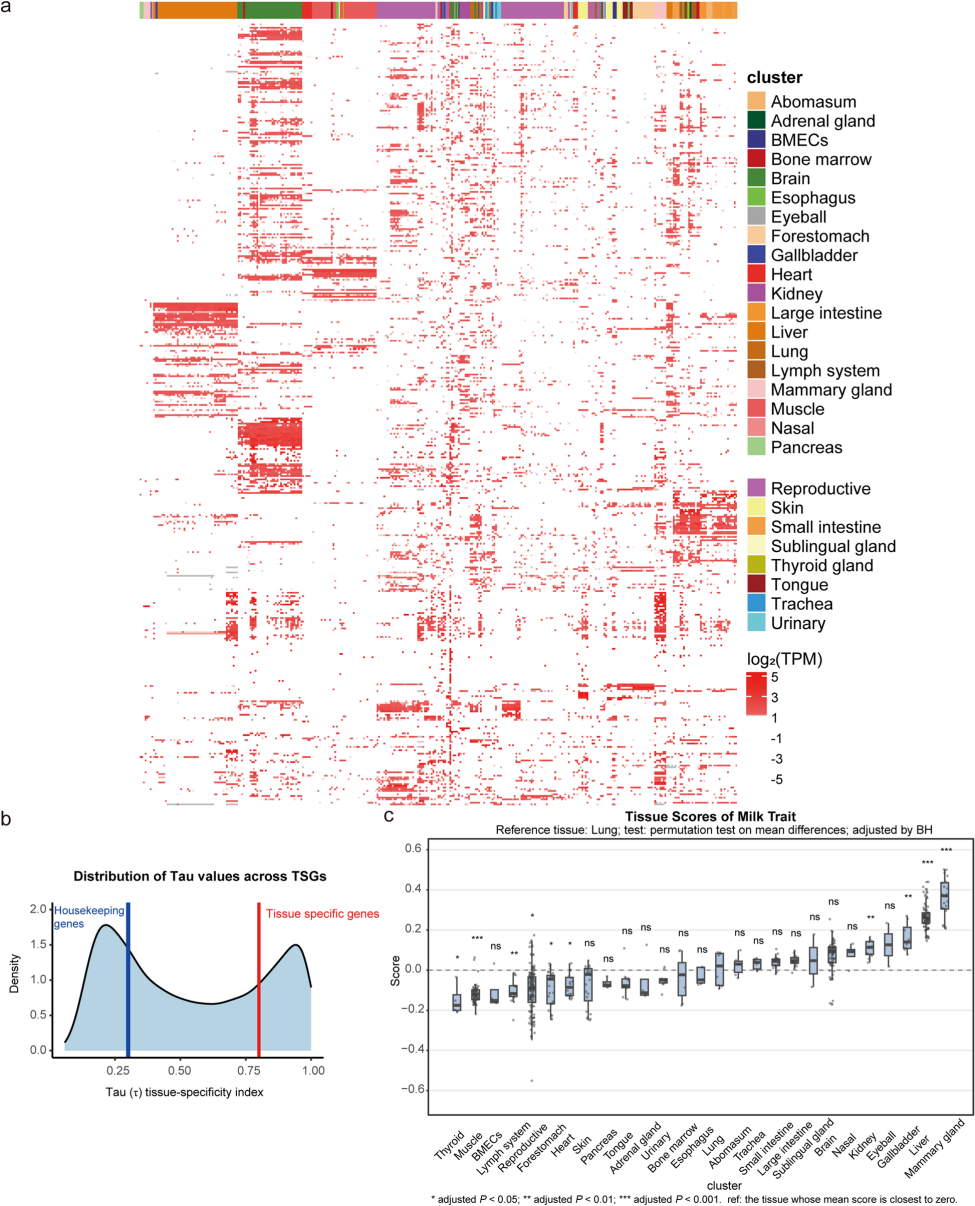
Tissue-driven transcriptomic organization and trait-relevant tissue prioritization. **a** Global gene expression landscape across bovine tissues based on all expressed genes. Samples cluster predominantly by tissue identity, indicating that transcriptomic variation is primarily driven by biological tissue differences rather than technical or individual effects. **b** Distribution of tissue specificity across the bovine transcriptome quantified by the tau index. Genes span a continuum from broadly expressed, housekeeping genes (tau < 0.3) to highly tissue-specific genes (tau > 0.8), enabling discrimination between conserved core transcriptional programs and tissue-restricted regulatory components. **c** Tissue-level module scores for milk yield trait. The mean scores of the mammary gland, liver, gallbladder, and kidney for milk yield were all significantly greater than 0 (*P* < 0.01), suggesting these tissues may represent important functional tissues underlying milk yield-related genetic signals

Subsequently, we constructed the most comprehensive pan-tissue transcriptomic profile of dairy cattle to date, encompassing 166 tissues and 23,899 high-quality genes. Genome-wide expression patterns revealed pronounced tissue specificity, with clustering largely reflecting biological function (Fig. 2a). Distinct expression profiles separated the brain and liver from other organs, while the small and large intestines, sharing similar physiological roles, clustered together. The reproductive system exhibited coherent tissue-specific expression as a distinct category. Moreover, approximately one-third of genes were preferentially expressed in specific tissues, indicating clear tissue-specific expression patterns (Fig. 2b).

### Tissue-specific expression patterns across bovine tissues

**Fig. 3:**
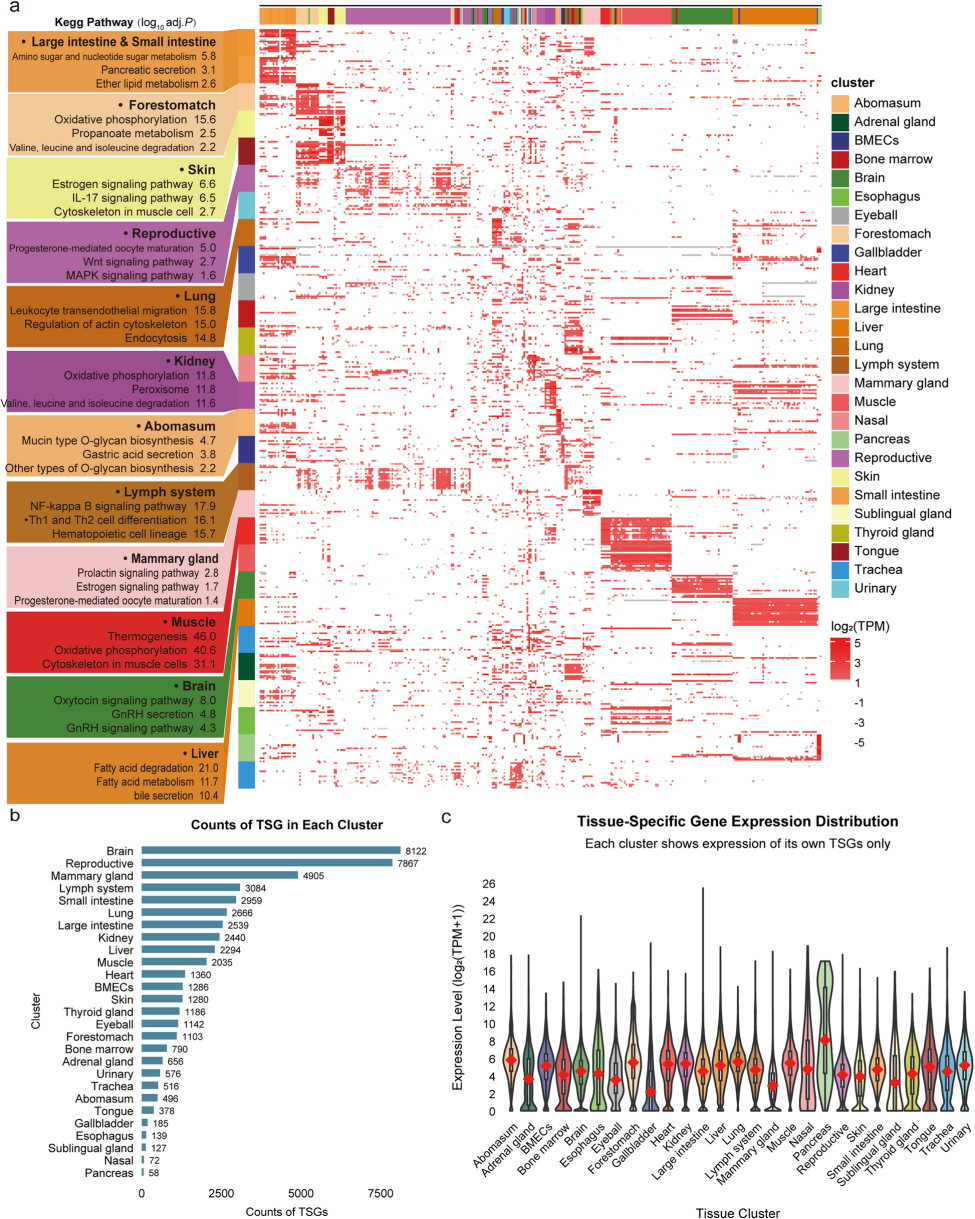
Tissue-specific genes recapitulate global tissue organization across bovine organs. **a** Heatmap of tissue-specific gene expression across all tissues. For each tissue, representative highly expressed tissue-specific genes were selected and visualized to construct the expression landscape. The resulting heatmap reveals pronounced tissue-driven clustering, indicating that tissue-specific genes alone are sufficient to distinguish tissue identity at the transcriptomic level. Functional annotations of major tissue-specific gene modules are shown on the left, and tissue cluster identities are indicated by color bars on the right. **b** Number of tissue-specific genes identified in each tissue cluster. **c** Tissue-specific genes show strong and selective expression in their corresponding tissues, consistent with their roles as core regulators of tissue-specific biological functions

To explore tissue-specific transcriptional programs and identify genes underpinning organ function, we compared each tissue against all others to define tissue-specific genes (log_2_FC > 1; adjusted *P* < 0.05). Across 166 tissues, the number of tissue-specific genes ranged from 58 to 8,122, with a mean of 1,861 per tissue. The pancreas exhibited the fewest tissue-specific genes, whereas the brain displayed the most. The variation in tissue-specific gene abundance reflected tissue metabolic demands and physiological specialization, with highly active tissues—including the brain, mammary gland, and reproductive organs—harboring larger numbers of tissue-specific genes (Fig. 3 and Table S2). Key tissue-specific gene profiles for representative tissues are summarized below.

#### Mammary gland

A total of 3,349 tissue-specific genes were identified in the mammary gland. Functional enrichment analysis revealed that these genes were predominantly involved in signaling pathways associated with epithelial differentiation, lactation, and hormonal regulation, including the prolactin, growth hormone, estrogen, and progesterone-mediated oocyte maturation pathways. Of them, several well-characterized milk-related genes such as *CSN1S1*, *CSN2*,* CSN3*,* CSN1S2*, and *ACACA* were included [19, 29–31]. Interestingly, *DGAT1*, a well-known major gene responsible for triglyceride synthesis in mammary epithelial cells [32], was not identified as a mammary tissue-specific gene.

Further, we implemented deconvolution analysis and obtained cell-type-level expression patterns of mammary gland based on the bovine single-cell transcriptomic atlas [10]. Through module scoring analysis, such 3,349 tissue-specific genes were shown highly expressed in luminal cells that are responsible for milk synthesis and hormonal response (Fig. S2a), indicating the lactation mechanism in mammary gland.

#### Liver

A total of 1,983 tissue-specific genes were identified in the liver. Functional enrichment analysis showed that liver tissue-specific genes were predominantly enriched in pathways related to fatty acid degradation, fatty acid and amino acid metabolism, bile secretion, and steroid and bile acid biosynthesis, consistent with the liver’s roles in energy metabolism and nutrient transport. Most liver tissue-specific genes were specifically expressed in hepatocytes. The expression level of liver-specific tissue-specific genes in hepatocytes is significantly higher than that in other cell types (Fig. S2b).

#### Rumen, reticulum, omasum and abomasum

Cattle, as typical ruminants, possess a forestomach composed of the rumen, reticulum, and omasum, among which the rumen harbors dense microbial populations and plays a dominant role in feed fermentation and nutrient absorption. Totally, 963 tissue-specific genes were jointly identified across the forestomach compartments, with significant enrichment in pathways related to oxidative phosphorylation, propanoate metabolism, and amino acid metabolism. The tissue-specific genes of rumen were predominantly expressed in spinous cells through deconvolution analysis based on single-cell atlas (Fig. S2c) [10], suggesting that nutrient metabolism and absorption may be primarily mediated by these functionally analogous cell types.

Further analysis of rumen subcompartments comprising dorsal sac, ventral sac, cranial sac and caudoventral blind sac, revealed distinct cellular expression patterns: tissue-specific genes in the dorsal sac were mainly expressed in pit-like and mucous cells; those in the cranial and caudoventral blind sacs were enriched in gastric muscle cells; and the ventral sac tissue-specific genes were primarily expressed in macrophages.

The abomasum, commonly known as the "true stomach" in ruminants, is primarily responsible for the secretion of gastric acid and digestive enzymes, which further break down the fermentation products from the rumen. Our analysis revealed that the tissue-specific genes in the abomasum were significantly enriched in the gastric acid secretion pathway, underscoring the critical role of this organ in acid production and digestive enzyme secretion. Among them, *ATP4A*, *ATP4B*, and *GAS*T [33–35] were strongly associated with gastric acid secretion, suggesting their vital involvement in the regulation of gastric acidity essential for digestion.

#### Intestine

The small intestine and large intestine exhibit significant similarities in their mRNA expression profiles, with 2,660 tissue-specific genes identified in the small intestine and 2,203 tissue-specific genes in the large intestine. The tissue-specific genes shared by the small and large intestines are primarily enriched in immune function-related pathways (adjusted *P* < 0.05), including FcγR-mediated phagocytosis, IgA immune network, antigen processing and presentation pathways and N-glycan biosynthesis. Tissue-specific genes of small intestine were significantly enriched in the pancreatic secretion pathway and bile secretion, amino sugar and nucleotide sugar metabolism, and ether lipid metabolism, tissue-specific genes of large intestine were significantly enriched in pathways for mineral absorption, bile secretion, and fat digestion and absorption, indicating the large intestine serves as a significant secondary site for the absorption of fatty acids, particularly short-chain fatty acids (SCFAs) produced by microbial fermentation.

Within the small intestine, the duodenal epithelium displayed digestive and absorption functions with significant enrichment in pathways like mineral absorption, insulin secretion, pancreatic secretion, cysteine and methionine metabolism, and nitrogen metabolism, indicating the role of the duodenum in regulating nutrient metabolism and digestive fluid secretion. Tissue-specific genes in the ileum were enriched in immune-related pathways such as c-type lectin receptor signaling, leukocyte transendothelial migration, and platelet activation, as well as pathways for cellular movement and structure, such as cytoskeleton in muscle cells, focal adhesion, regulation of actin cytoskeleton, and motor proteins, indicating its strong contractile and motility capabilities. Tissue-specific genes in the ileal mucosa were related to epithelial barrier maintenance and rapid cell turnover, such as polycomb repressive complex and ubiquitin-mediated proteolysis.

By integrating scRNA sequencing data [10], we found that the tissue-specific genes in the small intestine are predominantly expressed in progenitor cells and intestinal stem cells (Fig. S3a), effectively linking the nutrient absorption function of the small intestine. In the large intestine, the tissue-specific genes are primarily expressed in enterocyte cells (Fig. S3b).

#### Hypothalamus, pituitary, pineal and reproductive cluster

By clustering the identified tissue-specific genes, we observed a remarkable phenomenon that the hypothalamus, pituitary, and reproductive system tissues including ovary, corpus luteum, fallopian tube, uterus, and vagina were clustered in close spatial proximity, confirming their functional interconnections within the neuro-endocrine-reproductive axis. The brain tissue-specific genes were significantly enriched in classical endocrine regulatory pathways such as the GnRH signaling pathway, GnRH secretion pathway, oxytocin signaling pathway, and estrogen signaling pathway. Tissue-specific genes in reproductive tissues focused on progesterone-mediated oocyte maturation, MAPK and Wnt signaling pathways, representing downstream responses of target organs that maintain reproductive functions. Of note, we observed the tissue-specific genes (*VAX1*, *SIX6*, and *SIX3*) in hypothalamus involved in GnRH expression, neuronal migration, and the development of the hypothalamic reproductive axis [36–38]; *HESX1* and *PCSK1* for the processing and secretion of gonadotropic hormones were highly expressed in the pituitary [39, 40]; while in the ovary, the highly expressed genes *FSHR*, *ESR2*, *BMPR1B*, and *BMP15* are involved in executing ovarian functions, including gonadotropin responsiveness, follicular development, and steroid hormone regulation [41–44]. Together, these gene expression patterns form an integrated hypothalamic-pituitary-gonadal (HPG) axis that connects hormone synthesis, maturation, and target organ responses.

Further integrating scRNA sequencing data [10] of the cerebral cortex and cerebellum from Holstein cows, we found that the tissue-specific genes in the brain are highly expressed in excitatory neurons (Fig. S4a). The common tissue-specific genes in the ovary, uterine horn serosa, uterine body serosa and oviduct were highly expressed in pericytes cells (Fig. S4b), which may have the potential to participate in hormone-responsive tissue remodeling and vascular adaptation downstream of HPG-axis-related hormonal regulation [45].

### Tissue scoring

The tissue scoring result of milk yield trait showed that mammary gland, liver, gallbladder and kidney all had mean scores significantly greater than 0.0 (*P* < 0.01, Fig. 2c), suggesting these tissues may be more strongly associated with milk yield-related genetic signals. Similarly, for the other four traits, besides the mammary gland and liver, the brain and gallbladder also showed significantly positive scores (*P* < 0.01, Fig. S1), indicating potential tissue associations with these traits.

### Non-coding RNA expression patterns across bovine tissues

Non-coding RNAs (ncRNAs) play essential regulatory roles in gene expression by controlling transcription, mRNA stability, and translation [46–48].

#### MiRNA

To comprehensively characterize the bovine miRNA landscape, we analyzed small RNA sequencing data across tissues and generated an expanded miRNA catalog. In total, 1,008 miRNAs that expressed in at least 2 samples were identified, comprising 418 miRNAs annotated in miRBase and 590 newly identified miRNAs, substantially extending the current annotation of bovine miRNAs.

Among these miRNAs, 284 were broadly detectable, being identified in at least 90 tissues, whereas none exhibited restricted tissue distribution (fewer than 10 tissues). Despite this broad detectability, tissue-specificity analysis based on the tau index revealed that 942 miRNAs (93.45%) exhibited tau values greater than 0.8, demonstrating that the majority of miRNAs display strong tissue-biased expression patterns.

Family-level analysis revealed that the most abundant seed sequences—AAAAGUU/AAAACCU and AAAGUUC—corresponded to 42 miRNAs, of which 29 were assigned to the miR-2284/2285 family. These miRNAs displayed preferentially high expression in both primary bovine mammary epithelial cells (BMEC) and MACT, an immortalized bovine mammary epithelial cell line, indicating an expansion of the miR-2284/2285 family in mammary-related tissues and highlighting its potential relevance to mammary epithelial biology and milk-related traits.

**Fig. 4:**
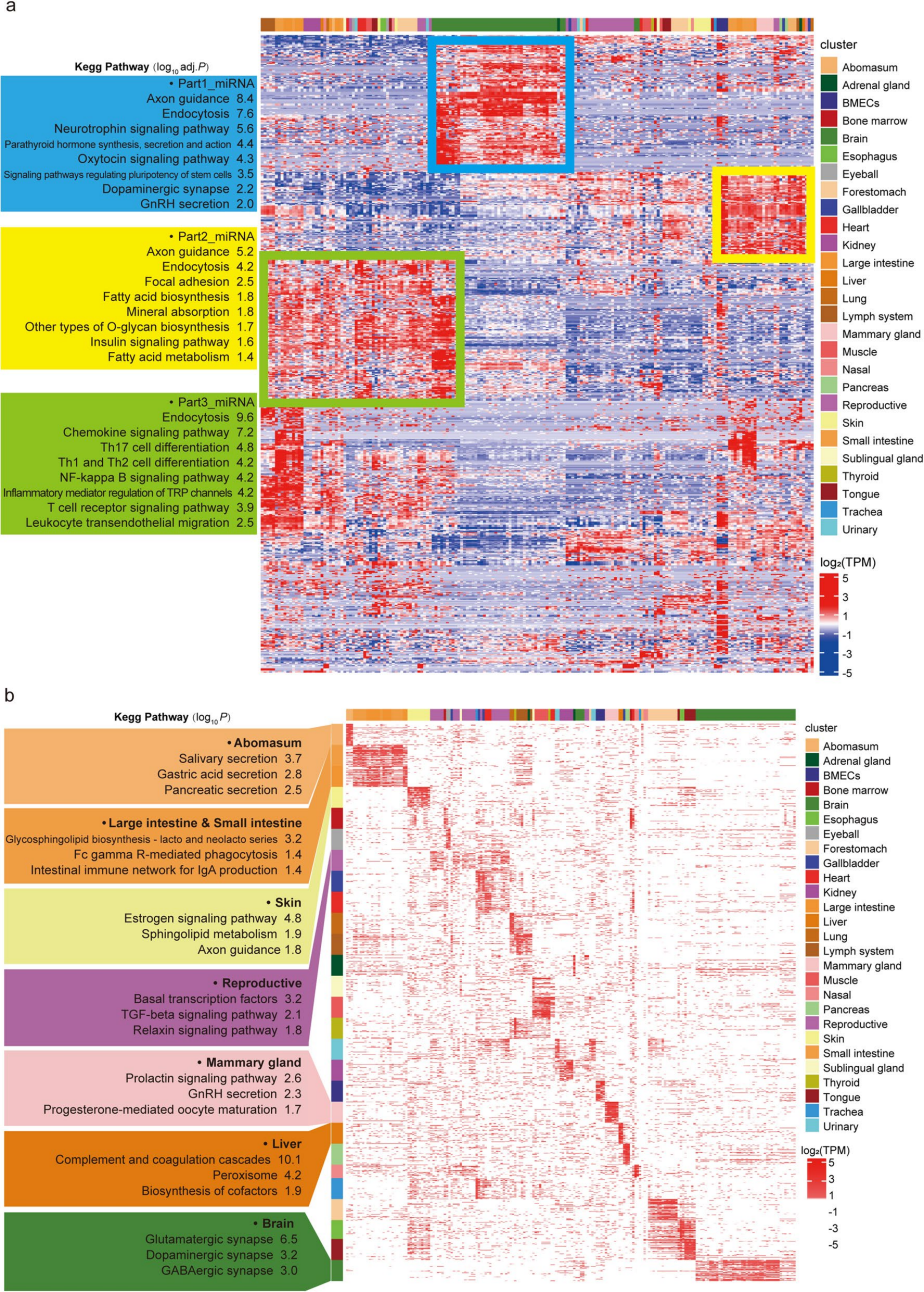
Tissue-specific miRNA and lncRNA expression landscapes reveal coordinated regulatory programs across bovine tissues. **a** Tissue-specific miRNA expression across tissues. A distinct cluster of miRNAs shows strong and preferential expression in brain tissues, including evolutionarily conserved miRNAs such as miR-219, miR-129, miR-653, and miR-329b, which have been previously implicated in neuronal differentiation, proliferation, and migration. Functional enrichment analysis of three major miRNA clusters is shown on the left, revealing cluster-specific biological associations, including immune-related pathways for one miRNA module. Tissue annotations are indicated on the right. **b** Heatmap of tissue-specific lncRNA expression across bovine tissues, demonstrating pronounced tissue-driven clustering patterns. Functional enrichment analysis of putative *cis*-regulated target genes of lncRNAs is shown on the left, highlighting tissue-relevant biological processes. Tissue annotations are shown on the right

To further explore tissue-associated miRNAs, we identified tissue-specific miRNAs across 27 tissue categories (log_2_ FC > 1; adjusted *P* < 0.05), with the number of tissue-specific miRNAs per tissue ranging from 0 (lung) to 246 (brain) (Table S4). The number of detected tissue-specific miRNAs showed a significant positive correlation with tissue sample size (*r* = 0.78, *P* < 0.01), highlighting the strong dependence of tissue-specific miRNA discovery on sample size. Notably, the brain exhibited a distinct tissue-specific miRNA expression pattern (Fig. 4a), characterized by enrichment of several evolutionarily conserved miRNAs, including miR-219, miR-129, miR-653, and miR-329b.

#### LncRNA

Using PLEK, CNCI, and LGC, we identified a total of 22,776 putative lncRNAs across 99 tissues. After read alignment and stringent quality control, a substantial fraction of reads exhibited ambiguous assignment and multi-mapping, resulting in zero quantifiable counts for many predicted lncRNAs. Consequently, 9,487 lncRNAs with measurable expression were retained for downstream analyses.

Among the quantified lncRNAs, 3,984 (41.99%) were detected in at least 90 tissues (n_tissue ≥ 90), indicating that broadly detectable lncRNAs constitute a considerable fraction of the dataset. At the tissue level, the number of expressed lncRNAs per tissue ranged from 4,676 in the pancreas to 8,337 in the superior vena cava. To further characterize tissue specificity, we computed the tau index for each lncRNA and found that a majority exhibited strong tissue-biased expression (tau > 0.8; 6,498 lncRNAs, 68.49%). Notably, widespread detectability and tissue bias are not mutually exclusive: by intersecting the broadly detected lncRNAs with the highly tissue-biased set, we identified 1,194 “ubiquitous-but-biased” candidates. The tissues in which these 1,194 lncRNAs reached their maximal expression spanned 94 tissues, without an obvious clustering pattern.

The results showed that the number of tissue-specific lncRNAs (log_2_FC > 1; adjusted *P* < 0.05) across tissues ranged from 32 to 2,403, with an average of 812 (Fig. 4b and Table S5). In contrast, the aorta exhibited a markedly higher number of tissue-specific lncRNAs than expected given its sample size (observed-to-expected ratio, O/E = 4.05). Overall, the heart-related cluster (aorta, superior vena cava, and left ventricle) contained 1,779 tissue-specific lncRNAs, among which the aorta contributed 1,315 of the most highly expressed lncRNAs. Functional enrichment analysis of the *cis*-target genes of these tissue-specific lncRNAs indicated that 241 out of 408 genes were annotated in KEGG, and the annotated set was significantly enriched in cytoskeleton-related pathways, consistent with the structural and contractile functions of vascular and cardiac tissues.

#### CircRNA

We identified a total of 209,368 unique circRNAs originating from 15,528 host genes across 99 tissues. CircRNA classification revealed a strong global bias toward exon–intron circular RNAs (EIciRNAs), which accounted for 85.90% of all detected circRNAs, whereas antisense circRNAs, transcribed from the strand opposite to their annotated host genes, constituted the second fraction of the circRNA repertoire.

At the expression level, circRNAs were generally detected at low abundance across tissues. To focus on circRNAs highly expressed among tissues, we applied a stringent abundance filter based on the top 10% of mean expression values (mean count ≥ 0.15), yielding 21,268 high-confidence circRNAs. The number of highly expressed circRNAs varied substantially among tissues, ranging from 2,517 in bovine mammary epithelial cells (BMEC) to 14,081 in the superior vena cava. Despite this variability, 1,378 circRNAs were detected in nearly all tissues (> 90), whereas only approximately 2% were restricted to fewer than ten tissues, indicating that most circRNAs exhibit broad tissue detectability rather than restricted expression.

**Fig. 5:**
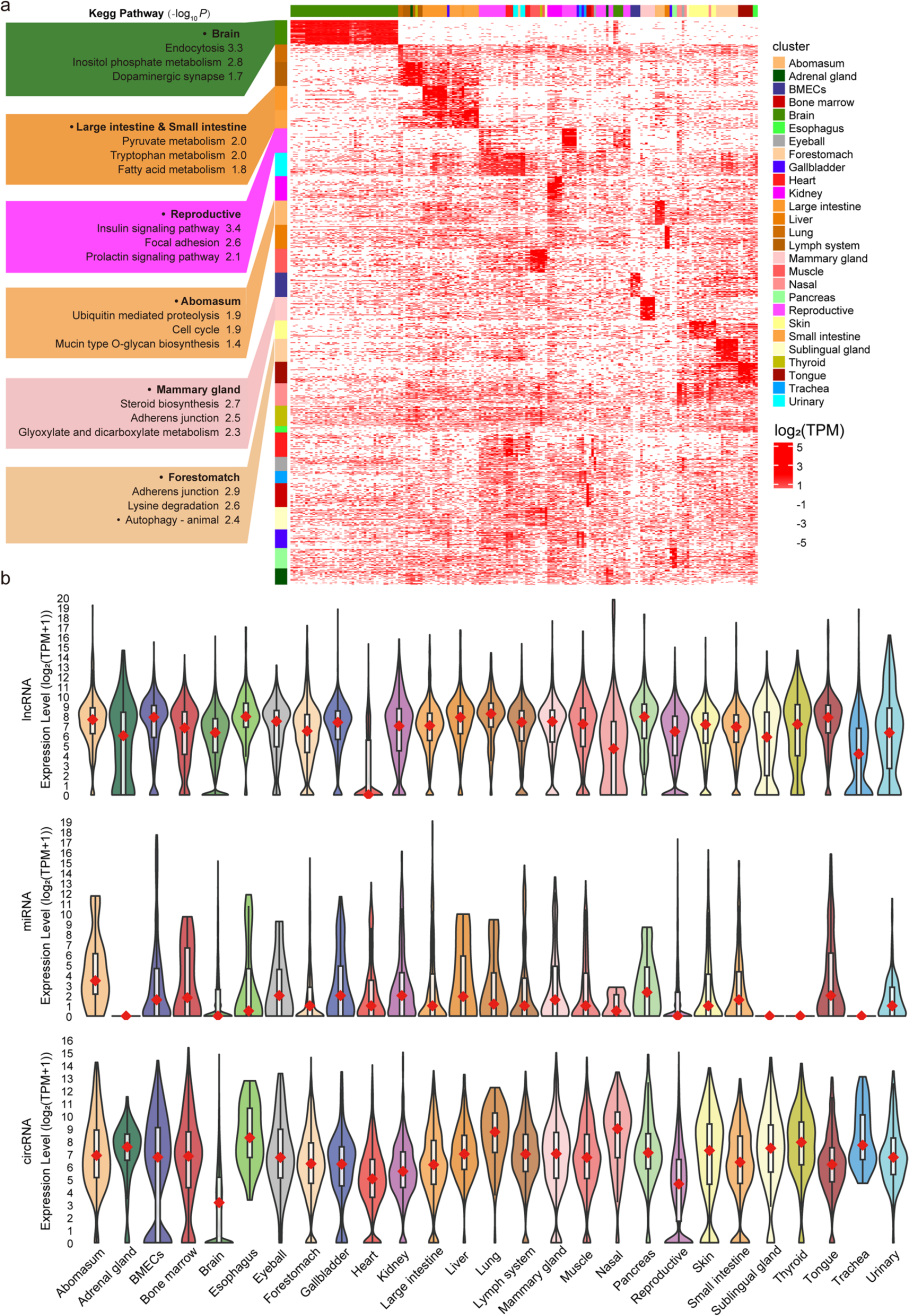
Tissue-specific expression landscape of circRNAs and comparative abundance of non-coding RNAs across bovine tissues. **a** Heatmap showing tissue-specific circRNA expression patterns across bovine tissues. CircRNAs exhibit pronounced tissue-specific expression and cluster primarily by tissue identity. Functional enrichment analysis of circRNA host genes is shown on the left, highlighting tissue-relevant biological processes, and tissue annotations are indicated on the right. **b** Comparison of expression abundance distributions among lncRNAs, circRNAs, and miRNAs across tissues, illustrating distinct global expression characteristics of different non-coding RNA biotypes

To identify tissue-specific circRNAs, we systematically compared each tissue against all others (log_2_FC > 0.5; adjusted *P* < 0.05). Across the 27 tissues analyzed, 2 to 289 tissue-specific circRNAs were detected per tissue (Fig. 5a and Table S6). The number of tissue-specific circRNAs exhibited a strong positive correlation with tissue sample size (Pearson *r* = 0.90), highlighting the sensitivity of tissue-specific circRNA discovery to sample size. Notably, all tissue-specific circRNAs were drawn from the high-expression circRNA set, indicating that tissue specificity is largely confined to robustly expressed circRNAs.

In the brain, tissue-specific circRNAs were derived from host genes significantly enriched for functions related to synaptic organization and excitatory neurotransmission. Among these, we highlight two genes with well-established roles in neuroendocrine regulation: *GPR158* and *GABRA1*. *GPR158* is a glucocorticoid-responsive receptor implicated in stress-related phenotypes and adrenal steroidogenesis [49], whereas *GABRA1* mediates GABAergic inhibition of corticotropin-releasing hormone (CRH) neurons in the hypothalamic paraventricular nucleus [50], thereby constraining CRH release and modulating downstream ACTH and glucocorticoid signaling.

#### CeRNA

The merged of RNAhybrid and miRanda yielded 184,554 miRNA–mRNA interactions (198 miRNAs and 10,421 mRNAs), 1,048,576 miRNA–lncRNA interactions (1,637 miRNAs and 7,884 lncRNAs), and 70,036,599 miRNA–circRNA interactions (1,713 miRNAs and 504,995 circRNAs).

In the mammary gland, we identified 110 matching pairs of tissue-specific lncRNA-miRNA, 30 matching pairs of tissue specific circRNA-miRNA, and 892 matching pairs of tissue-specific gene-miRNA. We then examined the regulatory miRNAs of two key genes, *CSN3* and *CSN1S1*. The regulatory miRNAs of these genes showed differential downregulation in the mammary gland (log_2_FC < −1), including 11 miRNAs such as miR-11972, miR-11994, and miR-204. These miRNAs together regulated 64 lncRNAs, including MSTRG.117537.3, and 10 circRNAs, including chr10:81387451-81388094.

### Alternative splicing events

**Fig. 6:**
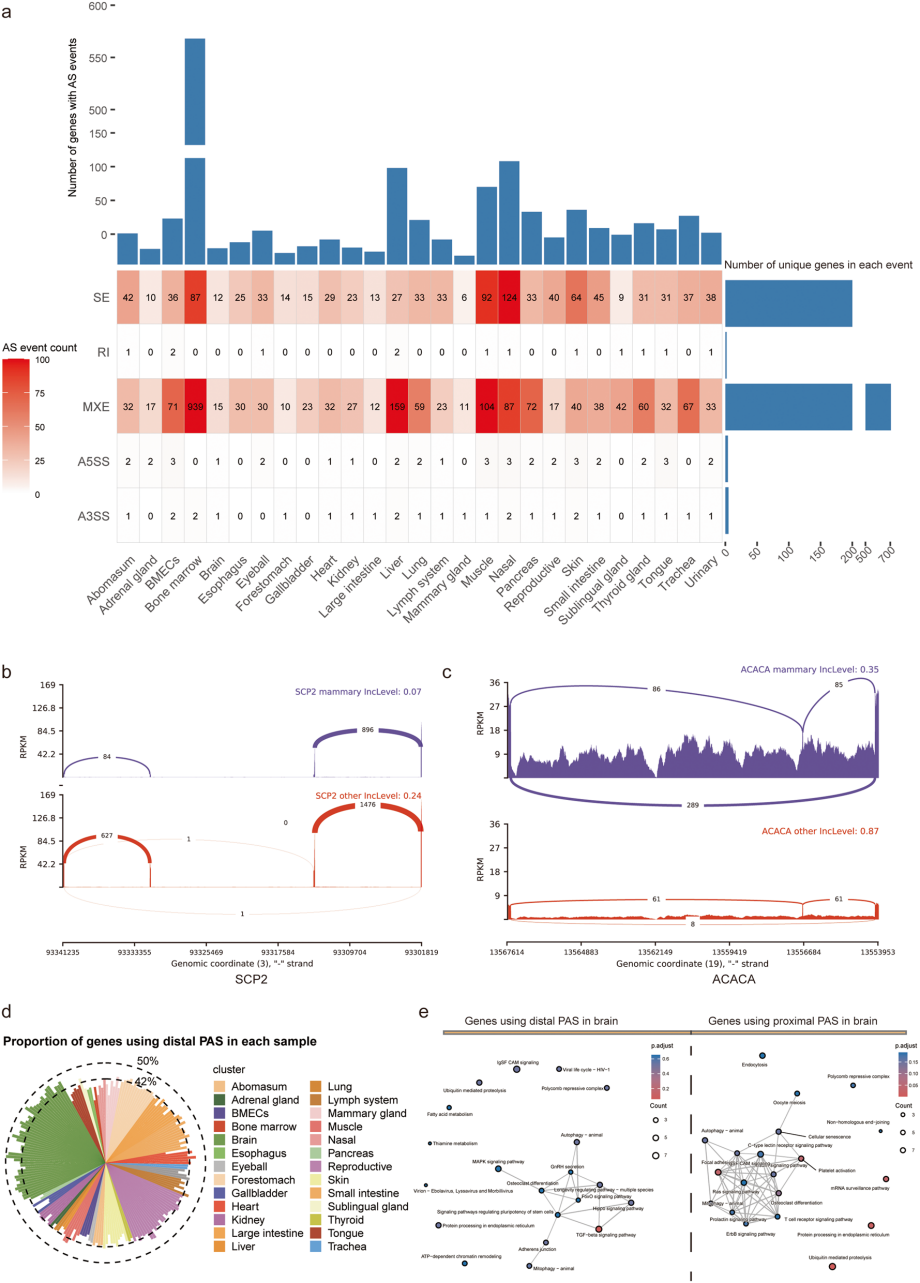
Landscape of post-transcriptional regulation across bovine tissues. **a** Overview of alternative splicing across tissues, showing the total number of alternative splicing events and the number of genes undergoing alternative splicing for each splicing type in each tissue. **b **and** c** Representative alternative splicing patterns of two mammary-relevant genes, *SCP2* and *ACACA*, illustrating tissue-specific exon usage and isoform composition. **d** Summary of alternative polyadenylation across tissues, showing the number of genes exhibiting alternative polyadenylation events and the distribution of distal poly(A) site usage index (PDUI). **e** Functional enrichment analysis of genes preferentially using distal or proximal poly(A) sites in the brain

Alternative splicing was widespread across bovine tissues and exhibited both shared and tissue-specific patterns. In total, we identified 105,859 AS events from 14,588 genes, including 34,943 mutually exclusive exon events from 9,671 genes, 1,071 retained intron events from 969 genes, 1,034 alternative 5′ splice site events from 828 genes, and 1,154 alternative 3′ splice site events from 933 genes (Fig. 6a and Table S7). After applying a read threshold of ≥ 50 to focus on robustly detected events, 4,409 genes with skipped exon (SE) events and 1,617 genes with MXE events were found to be shared across multiple tissues, indicating that AS contributes to common biological processes such as gene expression, cellular metabolism, and immune responses.

#### The most biologically informative AS patterns were observed in the mammary gland and liver

Among the analyzed tissues, the mammary gland and liver showed the most biologically informative AS patterns in relation to lipid metabolism. In the mammary gland, relatively few AS events were detected, but several genes associated with milk fat synthesis, lipid transport, and mammary epithelial function, including *PLIN2*, *ACACA*, *VPS13B*, *SCP2*, and *ABC*G2 [51–54], underwent tissue-specific splicing. Notably, *ABCG2*, *PLIN2*, and *ACACA* were also identified as mammary tissue-specific genes. *ACACA* displayed a clear tissue-dependent splicing pattern, whereas *SCP2* showed more pronounced isoform divergence (Fig. 6b). The dominant mammary *SCP2* isoform was predicted to encode a markedly shorter protein with substantial structural alteration, suggesting potentially important functional consequences. In the liver, AS events were more abundant and were significantly enriched in metabolic pathways central to liver function, including 2-oxocarboxylic acid metabolism, carbon metabolism, amino acid biosynthesis, fatty acid metabolism, and biosynthesis of unsaturated fatty acids. Notably, both *ACACA* and *SCP2* exhibited dominant isoform usage patterns distinct from those observed in the mammary gland. In particular, one *SCP2* splicing event overlapped with the alternatively spliced region identified in the mammary gland, suggesting that the same locus undergoes tissue-dependent isoform selection. Together, these findings suggest that some lipid metabolism-related genes exhibit similar alternative splicing patterns in the mammary gland and liver, but display distinct dominant transcript preferences between such two tissues.

#### AS in gastric tissues mainly pointed to two functional themes: smooth muscle contractility and lipid/nutrient processing

In the forestomach and abomasum, AS patterns were mainly associated with smooth muscle function and lipid handling. Within the forestomach compartments, we identified 14 SE and 10 MXE events, whereas the abomasum showed greater splicing complexity, with 42 SE and 32 MXE events. *TPM1* and *TPM3* underwent extensive AS events in both regions, consistent with the strong contractile demands of the gastric system [55]. In addition, several genes related to lipid metabolism and intracellular transport, including *OSBPL8*, *VPS13B*, *LPIN1*, and *PSAP*, displayed alternative splicing [56–59]. These results suggest that AS in gastric tissues may contribute to both contractility and the regulation of lipid metabolism and nutrient processing.

#### AS in the intestine converged on digestion, epithelial barrier maintenance, and motility

In the small intestine, we detected 38 MXE events across 23 genes and 45 SE events across 38 genes, whereas the large intestine exhibited comparatively reduced splicing activity (12 MXE events in 11 genes and 13 SE events in 13 genes). Functionally, these alternatively spliced genes converge on key aspects of intestinal physiology. *VPS13B* and *PSAP* are primarily associated with lipid digestion and transport, reinforcing the centrality of digestion along the intestinal tract. Structural and barrier integrity are supported by cytoskeleton- and junction-related genes, including *ADD3*, *MACF1 *(*ACF7*), *CLSTN1*, and *FLNA*, whose alternative splicing may modulate epithelial architecture and intercellular connectivity [60–62]. In parallel, *MFF* and *TPM3* contribute to intestinal motility and energy homeostasis by regulating mitochondrial dynamics and smooth muscle contraction, respectively [63, 64]. Intriguingly, *PSAP* and *TPM3* undergo both SE and MXE events in the small and large intestines, implying a versatile isoform repertoire that enables context-dependent regulation of intestinal metabolism, barrier function, and peristaltic activity.

### Alternative polyadenylation event analysis

Alternative polyadenylation profiles were analyzed on a per-tissue basis by quantifying genes exhibiting APA and calculating the usage ratio between proximal and distal polyadenylation sites. In total, 5,749 unique APA events from 2,712 genes were identified across all tissues (Fig. 6d). Among these genes, NFIA on chromosome 5 showed the highest number of valid APA events across tissues, with 3′UTR lengths ranging from 135 to 7,321 bp.

#### Distinct APA patterns in the mammary gland and liver

Distinct tissue-specific APA patterns were observed in the mammary gland and liver. In the mammary gland, 1,967 genes underwent 7,257 APA event occurrences, among which 3,425 occurrences favored proximal polyadenylation sites and showed a markedly higher average expression level (mean TPM = 6,345). These genes were mainly enriched in ubiquitin-mediated proteolysis, protein processing in the endoplasmic reticulum, axon guidance, and oocyte meiosis. By contrast, genes favoring distal polyadenylation sites showed lower average expression levels (mean TPM = 2,141) and were enriched in hormone- and lipid metabolism-related pathways, including prolactin signaling, progesterone-mediated oocyte maturation, adipocytokine signaling, and regulation of lipolysis in adipocytes. In the liver, 2,632 APA event occurrences were identified, including 1,250 occurrences favoring proximal polyadenylation sites (mean TPM = 5,220). These genes were enriched in ubiquitin-mediated proteolysis, adherens junction, and focal adhesion. In contrast, genes favoring distal polyadenylation sites showed higher expression levels (mean TPM = 6,481) and were enriched in immune- and repair-related pathways, including Hippo, Wnt, and Toll-like receptor signaling. Proximal polyadenylation sites usage of *IGF1R*, a gene associated with milk traits [65], was significantly higher in the mammary gland and liver than in other tissues (*P* = 0.001).

#### APA features in the abomasum and forestomach

In the rumen, 1,281 genes undergo 19,230 APA event occurrences, of which 8,604 preferentially utilize proximal polyadenylation sites, with a moderate average expression level (mean TPM = 3,890). These genes are enriched in pathways such as adherens junction, autophagy, and focal adhesion, highlighting their roles in epithelial renewal and mucosal barrier maintenance. In contrast, 5,597 occurrences favor distal polyadenylation sites (mean TPM = 3,534), and are enriched in core digestive pathways, including endocytosis, carbohydrate digestion and absorption, and fatty acid metabolism, suggesting a functional shift toward nutrient processing.

#### Extensive APA landscape in the brain

In the brain, 2,411 genes undergo 67,909 APA event occurrences, of which 27,788 preferentially utilize proximal polyadenylation sites (mean TPM = 2,602). These genes are enriched in pathways such as focal adhesion and signaling pathways regulating pluripotency of stem cells, implicating roles in neural development. In contrast, 21,706 occurrences favor distal polyadenylation sites (mean TPM = 2,632), and are enriched in synaptic pathways, including cholinergic and dopaminergic synapses, as well as diverse signaling cascades, highlighting their involvement in neuronal communication and functional specialization.

### Cross-species analysis

**Fig. 7:**
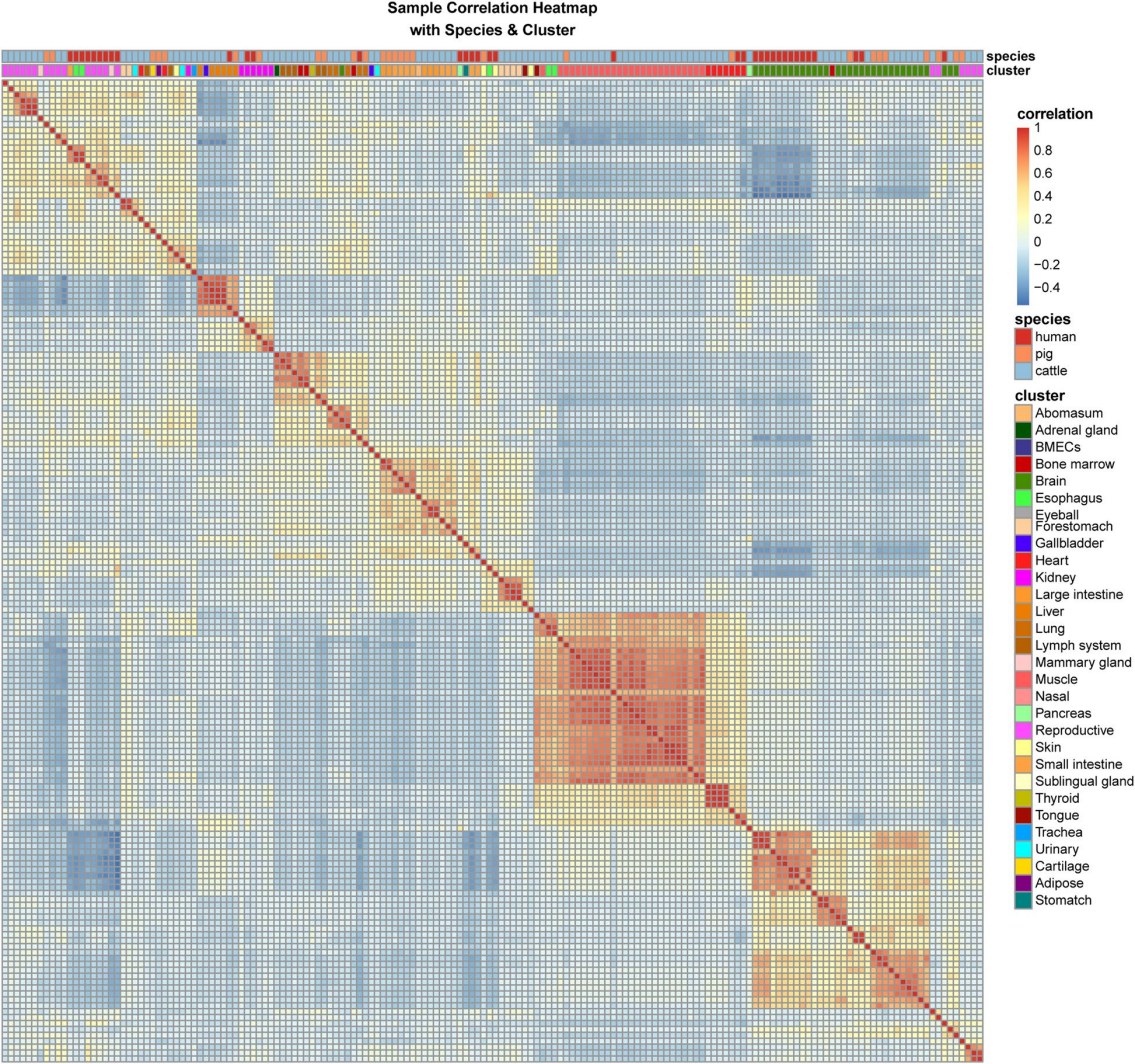
Cross-species correlation of tissue transcriptomes in cattle, pigs and humans. Heatmap shows pairwise correlations of tissue-averaged mRNA expression profiles across 166 bovine tissues, 22 porcine tissues and 35 human tissues. For each tissue, gene expression values were recalculated based on 11,547 one-to-one orthologous genes were averaged across biological replicates prior to computing correlations. The heatmap reveals that tissue identity generally outweighs species identity, with homologous tissues clustering together across mammals. Top annotations indicate species and tissue categories

Using 11,547 one-to-one orthologues shared among humans, pigs, and cattle, the cross-species heatmap revealed that for most tissues, tissue identity outweighed species identity, with homologous tissues from different species clustering together including brain, liver, kidney and others (Fig. 7).

An intriguing exception emerged within the reproductive system. Oviduct and ovarian-follicle samples in cattle, as well as testis and ovary in pigs, clustered closely with brain rather than with uterus. Conversely, the uterus clustered with the mammary gland. These results suggest that, beyond the classical gonads including ovary and testis, the oviduct exhibits an expression program more similar to tissues associated with the hypothalamic-pituitary-gonadal (HPG) axis, potentially acting as a secondary hormone-responsive organ.

A total of 1,935 one-to-one orthologs were identified from 3,656 bovine mammary tissue-specific genes, with 1,251 of these shared between cattle and humans. However, only 52 tissue-specific genes were conserved across all three species—humans, pigs, and cattle. This limited overlap likely stems from (i) pig dataset composition bias (285 adipose samples vs. 63 mammary samples), which dilutes mammary-specific expression signatures; and (ii) divergent breeding histories, since pigs have been selected for early weaning rather than sustained lactation, leaving mammary tissue under-selected. Human–cattle co-expressed tissue-specific genes were enriched in nuclear–cytoplasmic transport, PRC assembly, ubiquitin-mediated proteolysis, ATP-dependent chromatin remodeling, cell proliferation and differentiation, thyroid hormone signaling and parathyroid hormone synthesis. The 577 bovine mammary-specific genes were significantly enriched in PRC assembly and cell-cycle pathways. These results suggest that long-term dairy selection has reinforced the roles of conserved mammary tissue-specific genes in mammary gland, and the expansion of PRC assembly genes in bovine mammary tissue may underpin enhanced cell-cycle regulation necessary for persistent, rhythmic milk production.

## Discussion

This study constructed the most comprehensive pan-tissue transcriptomic profile of dairy cattle to date, covering 166 tissues and 23,899 high-quality genes. It systematically delineates the panoramic patterns of gene expression, non‑coding RNA regulation, alternative splicing, and alternative polyadenylation across tissues. The profile not only fills the gap left by previous transcriptomic studies largely limited to single or a few tissues, but also provides a multi‑layer and multi‑perspective molecular foundation for deciphering the cross‑tissue cooperative regulatory networks underlying important economic traits in dairy cattle, such as milk production.

### Tissue-specific gene expression underlying milk production and organ functional specialization

The tissue-specific expression patterns identified in the mammary gland and liver highlight their complementary roles in milk production. In the mammary gland, tissue-specific genes were enriched in pathways related to lactation, particularly milk protein synthesis and lipid metabolism, supporting its role as the direct functional organ of milk synthesis. The marked transcriptomic differences between cultured BMECs and in vivo mammary tissue further suggest that hormonal cues and cell–cell interactions are essential for maintaining the physiological state of the mammary gland, underscoring the value of organoid and 3D culture systems. By contrast, the liver functions primarily as a metabolic support organ, supplying precursors such as acetyl-CoA for de novo milk fat synthesis in the mammary gland and thereby supporting lactation through systemic metabolic coordination.

Distinct expression patterns were also observed among rumen subcompartments, indicating functional specialization in nutrient metabolism, absorption, and immune regulation. In particular, the ventral sac may be important for immune tolerance in a microbe-rich environment, whereas the dorsal sac may play a greater role in fatty acid uptake and transport. Tissue scoring further highlighted the multi-organ basis of milk production traits: in addition to the well-established roles of the mammary gland and liver, brain and bone tissues were positively associated with milk production traits, whereas muscle showed a negative association. Together, these findings suggest that milk production is shaped not only by the mammary gland itself, but also by coordinated regulation across multiple organs.

### Regulatory implications of non-coding RNAs across tissues

Distinct tissue-specific patterns were also observed for non-coding RNAs. Brain-enriched miRNAs, including miR-219, miR-129, miR-653, and miR-329b, have known roles in neuronal development and function, suggesting conserved regulatory roles in the bovine brain [66–69]. By contrast, lncRNAs with maximal expression were broadly distributed across tissues, indicating that lncRNA-mediated tissue-biased regulation may act across diverse biological systems. Notably, tissue-specific lncRNAs in the aorta may contribute to cardiovascular physiology through *cis*-regulation of genes such as *ITGA8*, *ITGA5*, and *CSRP1* [70–72]. CircRNAs provided another layer of regulatory complexity. Genes with large genomic spans and extensive exon content, such as the VPS13 family, appeared prone to generating multiple circRNA isoforms, suggesting that gene architecture may facilitate circRNA biogenesis. In addition, brain-enriched circRNAs may participate in neuroendocrine regulation, including the fine-tuning of hypothalamic-pituitary-adrenal axis activity. In the ceRNA networks, circRNAs accounted for the majority of miRNA-associated interactions [73–75], suggesting that they may serve as an important buffering layer for miRNA activity and fine-tune the expression of key genes, particularly in the mammary gland. Together, these findings support a coordinated non-coding RNA regulatory architecture involving miRNAs, lncRNAs, and circRNAs across tissues.

### Tissue-specific alternative splicing and functional diversification

This study reveals that the same gene exhibits distinct alternative splicing patterns across different tissues, which may reflect the varying physiological demands and functional differences of organs in fine-tuning biological processes. ACACA, which encodes the lipogenic enzyme ACC1 [76–79], provides a representative example: the mammary gland predominantly utilizes alternative isoforms, the brain favors the canonical transcript, and the liver expresses both isoforms at similar levels. This tissue-dependent isoform usage is consistent with the distinct roles of these organs in lipid metabolism, suggesting that alternative splicing may contribute to tissue-specific metabolic regulation.

### Alternative polyadenylation and its distinct regulatory effects across tissues

Alternative polyadenylation events can influence phenotypes by altering 3′ UTR length, thereby affecting mRNA stability, subcellular localization, translational efficiency, and ultimately protein output [15, 80]. In this study, alternative polyadenylation events in the mammary gland showed a clearer association with changes in gene expression than those in other tissues, suggesting that alternative polyadenylation in the mammary gland may regulate milk-related traits partly through miRNA-mediated control of gene expression. In contrast, in other tissues, alternative polyadenylation is more likely to affect phenotypes mainly through post-transcriptional regulation of transcript fate and protein output rather than through changes in total mRNA abundance.

### Limitations of this study

While this study provides a valuable multi-tissue transcriptomic resource, several limitations should be acknowledged. The newly generated multi-tissue data were derived from two adult Holstein cows, which limits the biological replication and population-level representativeness of the study. Furthermore, short-read sequencing techniques limit our ability to resolve full-length isoforms. Predicted regulatory interactions, such as those within ceRNA networks and alternative splicing events, require further experimental validation. To address these limitations, future studies incorporating long-read sequencing, expanded sample sizes, and single-cell atlases will enhance our understanding of tissue- and cell-type-specific regulation across diverse genetic backgrounds.

## Conclusions

By integrating 598 RNA sequencing datasets from 184 adult Holstein cows, we constructed a pan-tissue transcriptomic profile encompassing 166 tissues and 23,899 high-quality genes. We found that the identified tissue-specific genes revealed distinct tissue metabolic demands and physiological specialization, with highly active tissues (brain, mammary gland and reproductive organs) harboring larger numbers of tissue-specific genes. Such tissue-specific genes were regulated via extensive noncoding RNA interaction networks. At the post-transcriptional level, we observed that skipped exons and mutually exclusive exons were the predominant alternative splicing classes across tissues, and genes in the brain preferentially utilizes distal polyadenylation sites although proximal polyadenylation sites usage was widespread across tissues. Cross-species analysis among cattle, humans and pigs, indicated homologous tissues from different species clustered together in most tissues. Interestingly, oviduct was closely clustered with tissues within the HPG axis, potentially acting as a secondary hormone-responsive organ. These findings significantly contribute to understanding the mechanisms underlying complex trait formation and have substantial implications for cattle genetics, precision breeding, and potentially human health.

## Supplementary Information

Additional file 1: Table S1. Summary of mRNA sequencing datasets used in this study, including sample identifiers, tissue sources, sequencing platforms, read alignment rates, and other quality control metrics. Table S2. Milk production–related candidate genes retrieved from the Cattle QTL database, encompassing five milk production traits, including milk yield, fat yield, fat percentage, protein yield, and protein percentage. Table S3. List of tissue-specific genes identified across tissues. Genes were defined as tissue-specifically expressed based on a one-versus-all comparison using thresholds of log_2_FC > 1 and Benjamini–Hochberg adjusted *P* < 0.05. Table S4. List of tissue-specific miRNAs identified across tissues using DESeq2, with filtering criteria of log_2_FC > 1 and adjusted *P* < 0.05. Table S5. List of tissue-specific lncRNAs identified across tissues, defined by log_2_FC > 1 and adjusted *P* < 0.05. Table S6. List of tissue-specific circRNAs identified across tissues using DESeq2 (log_2_FC > 0.5, adjusted *P* < 0.05). Host gene annotations for each circRNA are provided. Table S7. Comprehensive list of alternative splicing events identified across all tissues using rMATS, including skipped exon (SE), mutually exclusive exon (MXE), alternative 5′ splice site (A5SS), alternative 3′ splice site (A3SS), and retained intron (RI) events. Differential alternative splicing events were defined using thresholds of |ΔPSI| ≥ 0.1 and Benjamini–Hochberg adjusted *P* < 0.05. Table S8. List of alternative polyadenylation events identified across tissues, including gene-level alternative polyadenylation events status and the dominant polyadenylation site (proximal or distal) in each tissue.

Additional file 2: Fig. S1. Tissue module scoring for four milk composition traits: fat yield (a), fat percentage (b), protein percentage (c), and protein yield (d). Fig. S2. Cell type scoring of mammary gland (a), liver (b), and rumen (c). Fig. S3. Cell type scoring of small intestine (a) and large intestine (b). Fig. S4. Cell type scoring of brain (a) and reproductive (b).

## Data Availability

The newly generated data in this study has been uploaded to the CNCB database, with the project number PRJCA060419. The sample numbers and metadata are updated in Table S1. The integrated data from BioProject accession numbers were PRJNA1017964, PRJNA263327, PRJNA589857, PRJNA844972, PRJNA856977, PRJNA714561, PRJNA573919, PRJNA357463, PRJNA907840, PRJNA475958, PRJNA517791 and PRJNA979929.

## Abbreviations

ACC1: Acetyl-CoA carboxylase 1
ACACA: Acetyl-CoA carboxylase alpha
ACADM: Medium-chain acyl-CoA dehydrogenase
ACTH: Adrenocorticotropic hormone
ATP4A: ATPase H^+^/K^+^ transporting alpha subunit
ATP4B: ATPase H^+^/K^+^ transporting beta subunit
BMEC: Bovine mammary epithelial cell
ceRNA: Competing endogenous RNA
circRNA: Circular RNA
CLSTN1: Calsyntenin 1
CRH: Corticotropin-releasing hormone
CSN1S1: Casein alpha S1
CSN1S2: Casein alpha S2
CSN2: Casein beta
CSN3: Casein kappa
CSRP1: Cysteine and serine rich protein 1
ESR1: Estrogen receptor 1
FDR: False discovery rate
GABRA1: Gamma-aminobutyric acid type A receptor subunit alpha1
GAST: Gastrin
GNB3: G protein subunit beta 3
GnRH: Gonadotropin-releasing hormone
GnRHR: Gonadotropin-releasing hormone receptor
GPR158: G protein-coupled receptor 158
GTEx: Genotype-tissue expression
GWAS: Genome-wide association study
HPA: Hypothalamic-pituitary-adrenal
HPG: Hypothalamic-pituitary-gonadal
lncRNA: Long non-coding RNA
ITGA5: Integrin alpha 5
ITGA8: Integrin alpha 8
INHBB: Inhibin beta B subunit
KEGG: Kyoto Encyclopedia of Genes and Genomes
miRNA: MicroRNA
mRNA: Messenger RNA
ncRNA: Non-coding RNA
NFIA: Nuclear factor 1 A-type
PCSK1: Proprotein convertase subtilisin/kexin type 1
PGR: Progesterone receptor
PLIN2: Perilipin 2
PRL: Prolactin
QTL: Quantitative trait locus
rMATS: RNA-Seq multivariate analysis of transcript splicing
SCP2: Sterol carrier protein 2
SNP: Single nucleotide polymorphism
SYT6: Synaptotagmin 6
ABCG2: ATP-binding cassette sub-family G member 2
TM6SF2: Transmembrane 6 superfamily member 2
UMAP: Uniform manifold approximation and projection
VPS13B: Vacuolar protein sorting 13 homolog B
