# Supplementary figures and images for: Pan-tissue transcriptomic profiling of dairy cattle
Source: J Anim Sci Biotechnol. 2026 Jun 14;17:120. doi: 10.1186/s40104-026-01440-9 (PMC13264825; doi:10.1186/s40104-026-01440-9)

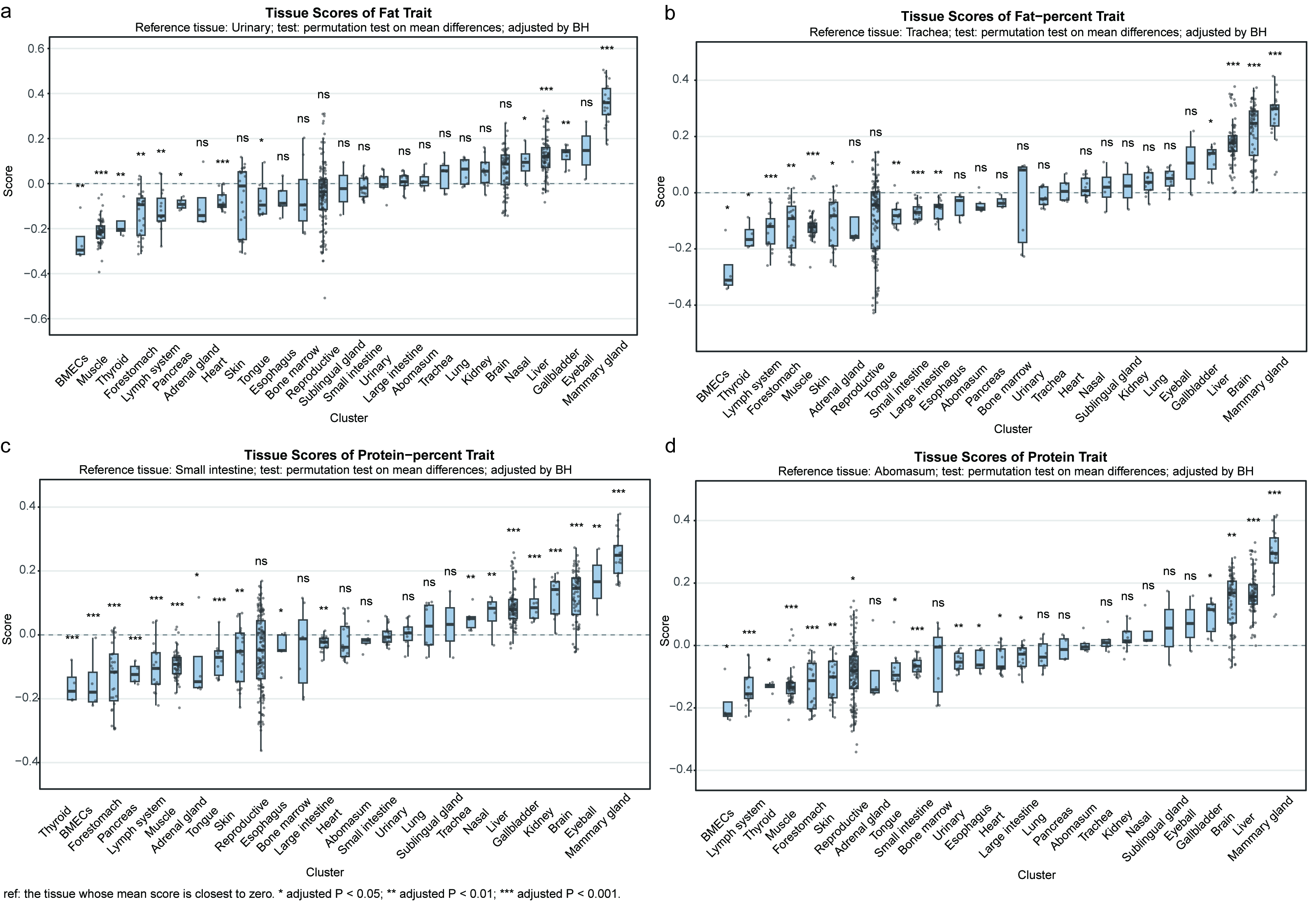

Supplement: Supplementary file 2 — Additional file 2: Fig. S1. Tissue module scoring for four milk composition traits: fat yield (a), fat percentage (b), protein percentage (c), and protein yield (d). Fig. S2. Cell type scoring of mammary gland (a), liver (b), and rumen (c). Fig. S3. Cell type scoring of small intestine (a) and large intestine (b). Fig. S4. Cell type scoring of brain (a) and reproductive (b). [file 40104_2026_1440_MOESM2_ESM.zip › Fig S1.tif]

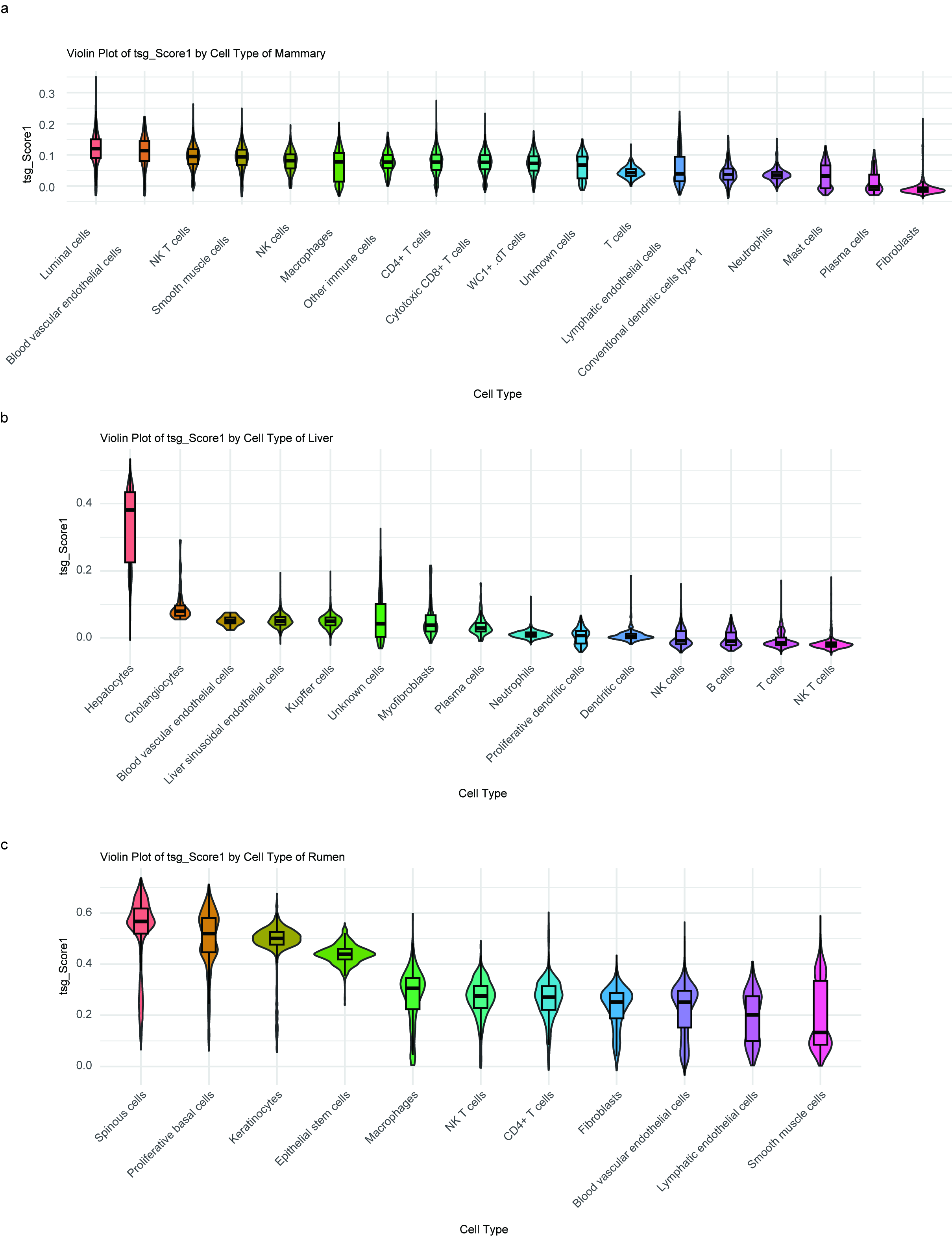

Supplement: Supplementary file 2 — Additional file 2: Fig. S1. Tissue module scoring for four milk composition traits: fat yield (a), fat percentage (b), protein percentage (c), and protein yield (d). Fig. S2. Cell type scoring of mammary gland (a), liver (b), and rumen (c). Fig. S3. Cell type scoring of small intestine (a) and large intestine (b). Fig. S4. Cell type scoring of brain (a) and reproductive (b). [file 40104_2026_1440_MOESM2_ESM.zip › Fig S2.tif]

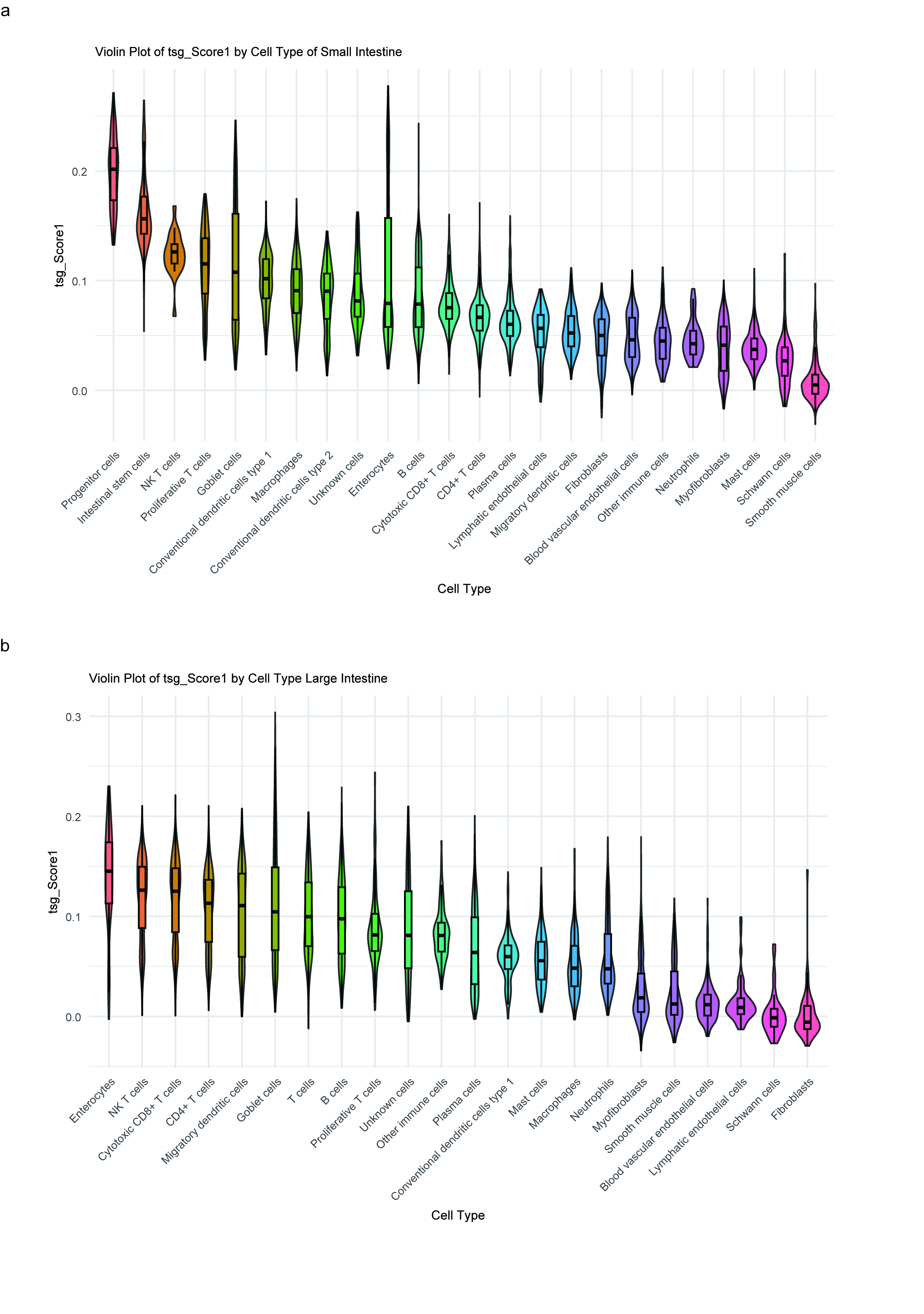

Supplement: Supplementary file 2 — Additional file 2: Fig. S1. Tissue module scoring for four milk composition traits: fat yield (a), fat percentage (b), protein percentage (c), and protein yield (d). Fig. S2. Cell type scoring of mammary gland (a), liver (b), and rumen (c). Fig. S3. Cell type scoring of small intestine (a) and large intestine (b). Fig. S4. Cell type scoring of brain (a) and reproductive (b). [file 40104_2026_1440_MOESM2_ESM.zip › Fig S3.tif]

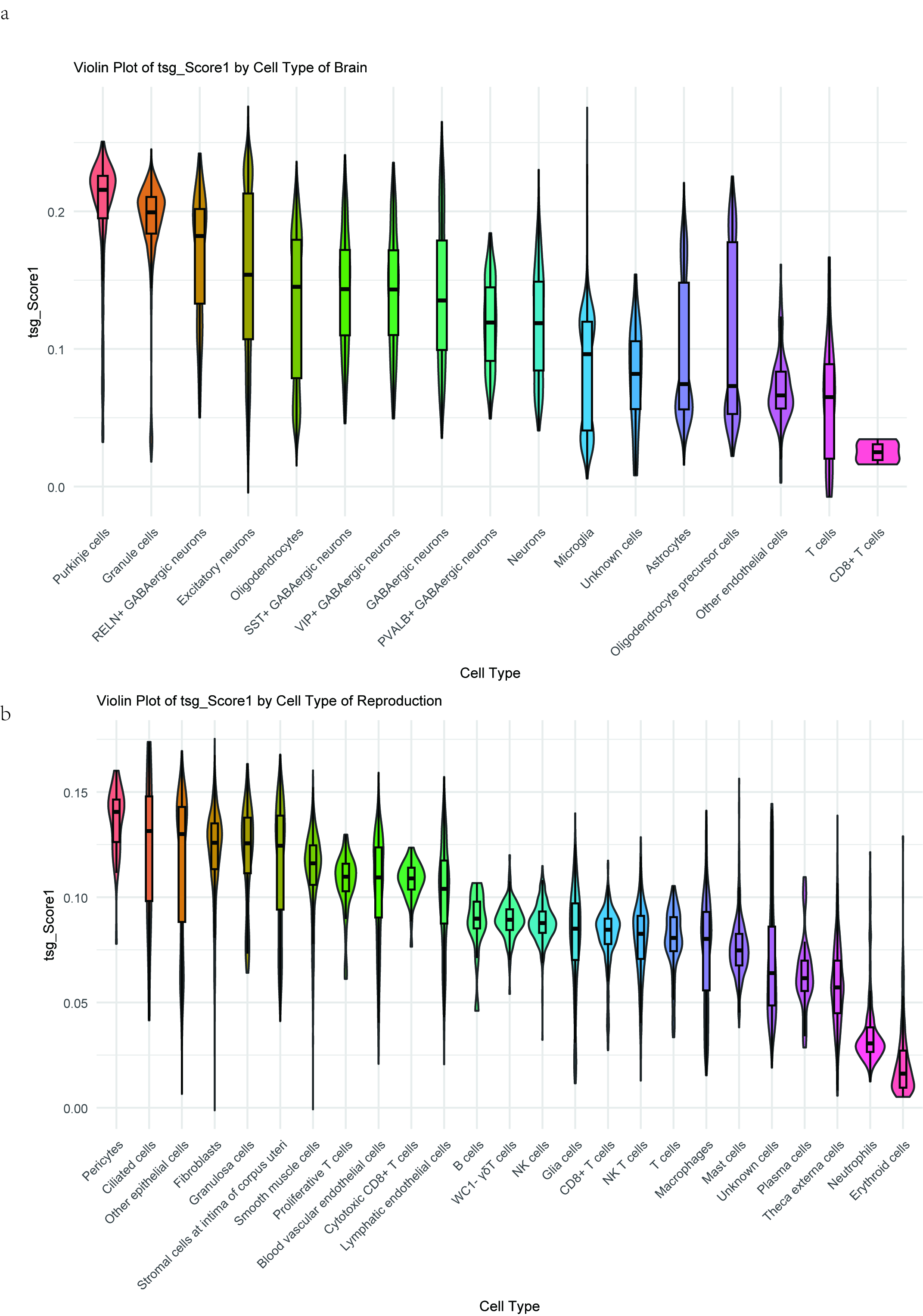

Supplement: Supplementary file 2 — Additional file 2: Fig. S1. Tissue module scoring for four milk composition traits: fat yield (a), fat percentage (b), protein percentage (c), and protein yield (d). Fig. S2. Cell type scoring of mammary gland (a), liver (b), and rumen (c). Fig. S3. Cell type scoring of small intestine (a) and large intestine (b). Fig. S4. Cell type scoring of brain (a) and reproductive (b). [file 40104_2026_1440_MOESM2_ESM.zip › Fig S4.tif]
